# Clathrin Assembly Protein CALM Plays a Critical Role in KIT Signaling by Regulating Its Cellular Transport from Early to Late Endosomes in Hematopoietic Cells

**DOI:** 10.1371/journal.pone.0109441

**Published:** 2014-10-03

**Authors:** Shinya Rai, Hirokazu Tanaka, Mai Suzuki, Honami Ogoh, Yasuhiro Taniguchi, Yasuyoshi Morita, Takahiro Shimada, Akira Tanimura, Keiko Matsui, Takafumi Yokota, Kenji Oritani, Kenji Tanabe, Toshio Watanabe, Yuzuru Kanakura, Itaru Matsumura

**Affiliations:** 1 Department of Hematology and Rheumatology, Kinki University Faculty of Medicine, Osaka, Japan; 2 Division of Hematological Malignancy, National Cancer Center Research Institute, Tokyo, Japan; 3 Department of Hematology and Oncology, Osaka University Graduate School of Medicine, Osaka, Japan; 4 Medical Research Institute, Tokyo Women’s Medical University, Tokyo, Japan; 5 Department of Biological Science, Graduate School of Humanities and Sciences, Nara Women’s University, Nara, Japan; Feinberg Cardiovascular Research Institute, Northwestern University, United States of America

## Abstract

CALM is implicated in the formation of clathrin-coated vesicles, which mediate endocytosis and intracellular trafficking of growth factor receptors and nutrients. We previously found that *CALM*-deficient mice suffer from severe anemia due to the impaired clathrin-mediated endocytosis of transferrin receptor in immature erythroblast. However, CALM has been supposed to regulate the growth and survival of hematopoietic stem/progenitor cells. So, in this study, we focused on the function of CALM in these cells. We here show that the number of Linage^−^Sca-1^+^KIT^+^ (LSK) cells decreased in the fetal liver of *CALM* ^−/−^ mice. Also, colony forming activity was impaired in *CALM^−/−^* LSK cells. In addition, SCF, FLT3, and TPO-dependent growth was severely impaired in *CALM^−/−^* LSK cells, while they can normally proliferate in response to IL-3 and IL-6. We also examined the intracellular trafficking of KIT using *CALM* ^−/−^ murine embryonic fibroblasts (MEFs) engineered to express KIT. At first, we confirmed that endocytosis of SCF-bound KIT was not impaired in *CALM* ^−/−^ MEFs by the internalization assay. However, SCF-induced KIT trafficking from early to late endosome was severely impaired in *CALM* ^−/−^ MEFs. As a result, although intracellular KIT disappeared 30 min after SCF stimulation in wild-type (WT) MEFs, it was retained in *CALM* ^−/−^ MEFs. Furthermore, SCF-induced phosphorylation of cytosolic KIT was enhanced and prolonged in *CALM* ^−/−^ MEFs compared with that in WT MEFs, leading to the excessive activation of Akt. Similar hyperactivation of Akt was observed in *CALM* ^−/−^ KIT^+^ cells. These results indicate that CALM is essential for the intracellular trafficking of KIT and its normal functions. Also, our data demonstrate that KIT located in the early endosome can activate downstream molecules as a signaling endosome. Because KIT activation is involved in the pathogenesis of some malignancies, the manipulation of CALM function would be an attractive therapeutic strategy.

## Introduction

Clathrin-mediated endocytosis (CME) is an active cellular process for membrane trafficking, which mediates the entry of nutrients and growth factor receptors into the cells [1]. When the target molecule binds to plasma membrane receptor, clathrin and accessory molecules such as AP2, epsin, clathrin assembly lymphoid myeloid leukemia protein (CALM) are recruited from cytoplasm into the membrane, resulting in the formation of clathrin-coated vesicles. Clathrin-coated vesicles transport target molecules from the membrane to early and late endosomes. Also, it regulates the intercellular trafficking among endosomes, trans-Golgi network (TGN), and lysosomes, thereby regulating the degradation of the target molecules [1]–[3].

Receptor tyrosine kinases (RTKs) such as KIT, epidermal growth factor receptor (EGFR) and MET play crucial roles in the development and maintenance of the cells, on which they are expressed [4]–[7]. Upon the ligand binding, activated RTKs are internalized and transferred to early endosome. Then, clathrin-coated RTKs are partially sorted back to the plasma membrane via recycling endosome (RE). The remaining RTKs are transported to the intraluminal vesicles (ILVs) of late endosomes/multivesicular body (MVB). MVB can be fused to the lysosomes, where RTKs are degraded [8]. Previously, it was believed that several signaling molecules such as MAPKs, JAK/STATs, and PI3K/AKT are activated by RTKs on the plasma membrane. However, several recent reports showed that these signaling molecules can also be activated by clathrin-coated RTKs located in the endosome, leading to the establishment of the concept “signaling endosome” [9]–[11].

*CALM* encodes a 652 aa protein with multiple domains such as AP180 N-terminal homology (ANTH) domain, DPF motif, NPF motif, and type I and II clathrin-binding sequences (CBS I and II), of which expression is ubiquitously observed in various organs [12]–[18]. Knockdown of CALM by RNA interference leads to the formation of larger and more irregular, clathrin-coated vesicles in HeLa cells, indicating that CALM is required for proper formation of clathrin-coated vesicles [19]. *CALM* was originally isolated as a part of the fusion gene *CALM/AF10*, which results from the chromosomal translocation t(10;11) (p13;q14) [20]. This translocation is found in acute lymphoblastic leukemia (ALL), acute myeloid leukemia (AML) and malignant lymphomas [21]. Also, it was shown that overexpression of *CALM/AF10* in primary murine bone marrow (BM) cells resulted in the development of an aggressive form of leukemia in a murine BM transplantation model [22], [23]. These results suggest that CALM would play an important role in the growth and differentiation of hematopoietic cells. This hypothesis was subsequently supported by the reports that *fit1* mutants, which contain nonsense point mutations in the *CALM* gene [24], [25]. In these mice, the number of early hematopoietic progenitor cells was severely reduced and numerous morphologic and functional defects were observed in the peripheral blood. However, detailed analysis on the hematopoietic defects in *fit1* mutants has not been performed.

To clarify the physiological role of CALM *in* *vivo*, we recently generated *CALM*-deficient mice [26]. Although *CALM* ^+/−^ mice didn’t shown an apparent phenotype, *CALM* ^−/−^ mice exhibited retarded growth *in* *utero* and were dwarfed throughout their shortened life-spans. Moreover, *CALM*-deficient mice suffered from severe anemia due to the impaired CME of transferrin in immature erythroblast. Meanwhile, based on the report of *fit1* mutants [24], [25], CALM has been supposed to regulate the growth and survival of hematopoietic stem/progenitor cells. So, in this study, we focused on the molecular mechanism through which CALM regulates their growth and survival. We here show that KIT-mediated growth was impaired in *CALM*-deficient hematopoietic stem/progenitor cells, probably due to the defect in the KIT trafficking from early to late endosomes.

## Materials and Methods

### Ethics Statement

This study was approved by the Committee of Animal Experiments, Kinki University Faculty of Medicine (approval ID: 06-13).

### Recombinant Growth Factors and Inhibitors

Recombinant murine stem cell factor (SCF), Flt-3 ligand (FL), thrombopoietin (TPO), interleukin-3 (IL-3), and IL-6 were purchased from PeproTech. (Rocky Hill, NJ). Imatinib mesylate (STI571) purchased from Selleck (Houston, TX) was used to inhibit KIT activity and Bafilomycin A1 purchased from Sigma Aldrich (St. Louis, MO) was used to inhibit the protein transport from early to late endosome [27].

### Isolation and Immortalization of Murine Embryonic Fibroblasts (MEFs)

Primary MEFs were isolated from wild-type (WT) and *CALM*-deficient mice on embryonic day 14.5 (E14.5) and cultured in Dulbecco’s modified Eagle’s medium (DMEM) supplemented with 15% fetal calf serum (FCS) and 5% CO2 at 37°C. To immortalize MEFs, we transfected the expression vector for SV40 large T antigen into MEFs by Lipofectamine 2000 Reagent (Invitrogen, Life Technologies, Carlsbad, CA) according to the manufacturer’s instructions. Stable immortalized clones were obtained by serial dilution.

### Retrovirus Transduction

Murine full-length *Kit* cDNA kindly provided from Dr. Mizuki M. (Osaka University, Osaka, Japan) was subcloned into pMSCV-IRES-EGFP bicistronic retrovirus vector. The retrovirus vector was transfected into a packaging cell line 293T containing the expression plasmids for *gag* and *pol*, which was cultured in DMEM supplemented with 10% FCS. The supernatant was collected 48 h after transfection. MEFs were plated onto the 3.5 cm dish coated with fibronectin fragment (RetroNectin, Takara Bio, Shiga, Japan) and cultured with 1 ml virus supernatant for 72 h. Retrovirus-infected MEFs were isolated as GFP-positive cells by FACS Aria (BD Biosciences, San Jose, CA).

### Purification of Murine Lineage^−^ Sca-1^+^ KIT^+^ (LSK) Cells

Murine fetal liver cells were harvested from E14.5 embryos and mononuclear cells (MNCs) were separated by density gradient centrifugation. Then, MNCs were incubated with the antibodies (Abs) as follows: anti-lineage Abs (a cocktail of biotinylated Abs against CD3e (145–2C11), CD45R/B220 (RA3–6B2), Gr-1 (RB6–8C5), and TER-119 (TER-119)), fluorescein isothiocyanate (FITC)-conjugated anti-Sca-1 Ab (D7), allophycocyanin (APC)-conjugated anti-c-KIT Ab (2B8), and streptavidin-phycoerythrin (PE)-cyanin (cy)7 (BD Biosciences). After staining, LSK cells were sorted by FACS Aria. Non-viable cells were eliminated by the staining with 7-amino-actinomycin D (Calbiochem, Merck Millipore, Darmstadt, Germany).

### Clonogenic Assay

LSK cells from WT or *CALM^−/−^* mice were plated onto Complete Medium with murine Cytokines MethoCult GF M3434 (StemCell Technologies, Vancouver, BC, Canada). The numbers of colony forming unit–mixed (CFU-Mix), CFU-granulocyte macrophage (CFU-GM), and burst-forming unit–erythroid (BFU-E) were counted under the inverted microscope 14 days after plating.

### Proliferation Assay

LSK cells were cultured in Roswell Park Memorial Institute-1640 (RPMI-1640) medium (Gibco, Life Technologies, Carlsbad, CA) with 10% FCS containing 100 ng/ml, SCF, 100 ng/ml FL, 100 ng/ml TPO, 100 ng/ml IL-3, 100 ng/ml IL-6 at 37°C. Number of viable LSK cells was measured by the Cell Titer Glo Reagent (Promega, Madison, WI) from the intensity of the luminescence using an Envision plate reader (1420 ARVO MX-2, Wallac, PerkinElmer, Inc., Waltham, MA).

### Flow Cytometric Analysis

The expression of surface molecules was examined by FACS Aria using the appropriate Abs and these results were analyzed by BD FACS Diva software (BD Biosciences) or FlowJo software (TreeStar, Ashland, OR). To analyze cytoplasmic AKT phosphorylation in KIT^+^ hematopoietic cells by flow cytometry, MNCs isolated from BM were fixed with 3.7% (w/v) formaldehyde in PBS for 15 min, and permeabilized with 1% (w/v) bovine serum albumin (BSA) and 0.1% (v/v) Triton X-100 in PBS for 15 min. These cells were incubated with the Alexa647-conjugated anti-phosphorylated Akt Ab (Cell Signaling Technology, Danvers, MA) in combination with the Abs to identify KIT^+^ cells as described above. After staining, these cells were analyzed by FACS Aria.

### Internalization Assay

Internalization assay for KIT was performed as described previously [26]. Briefly, WT and *CALM^−/−^* MEFs both engineered to express KIT were cultured with biotinylated SCF (R&D systems, Minneapolis, MN) for 60 min, and further incubated with the APC-conjugated streptavidin (Biolegend, San Diego, CA) for 30 min at 4°C. Then, these cells were incubated at 37°C up to 20 min to allow internalization. After stripping unincorporated SCF with acidic buffer (20 mM MES pH 5, 130 mM NaCl, 2 mM CaCl2 and 0.1% BSA), relative amount of internalized SCF-KIT complex was evaluated from the fluorescence intensity by FACS at the indicated times compared with the initial amount of membrane KIT.

### Immunofluorescence Analysis

*KIT*-transfected MEFs were transferred onto the coverslips and cultured in DMEM supplemented with 15% FCS at 37°C for 48 h. After the stimulation with 100 ng/ml SCF for the indicated times, the cells were washed with ice-cold PBS and then fixed with 3.7% (w/v) formaldehyde in PBS for 15 min. After incubation in blocking buffer with 1% (w/v) bovine serum albumin (BSA) and 0.1% (v/v) Triton X-100 in PBS for 15 min, the fixed cells were incubated with the primary and then with the secondary Abs suspended in the reagents each for 45 min. The utilized Abs and reagent were as follows: biotinylated anti-KIT (2B8, Biolegend), anti-Rab5 (C8B1), anti-Rab7 (D95F2), anti-Rab11 (D4F5) Abs (Cell Signaling Technology), AlexaFluor 488-conjugated anti-rabbit IgG Ab, AlexaFluor 568 streptavidin conjugates (Invitrogen). After washing with PBS, the coverslips were mounted on glass slides using Prolong Gold antifade reagent (Invitrogen) and observed under the confocal microscopy (LSM-410, Nikon, Tokyo, Japan).

### Subcellular Protein Fractionation

Subcellular protein fractionation was performed using OptiPrep density gradient medium (Axis-Shield, Oslo, Norway) following their protocol (S23) with some modification. Briefly, cell lysates were centrifuged at 1000 g for 5 min to pellet nuclei and cell debris. Supernatant was loaded on the Opti-Prep discontinuous gradient (30, 25, 20, 15, 10, 5%) and centrifuged at 90,000 g for 16 h. After centrifugation, 50 fractions were collected and subjected to immunoblot analyses.

Subcellular protein fractionation was also performed with a subcellular protein fractionation kit (Thermo scientific, Waltham, MA). Briefly, the cultured cells were incubated in DMEM supplemented with 15% FCS in the presence of 25 µg/ml cycloheximide (Cell Signaling Technology) at 37°C for 1 h, and stimulated with 100 ng/ml SCF for the indicated times. Cultured cells were washed with ice-cold PBS and centrifuged. The pellet was suspended in cytoplasmic extraction buffer at 4°C for 10 min and centrifuged at 500 g for 5 min. The supernatant was transferred and used as a cytoplasmic extract. Then, the remaining pellet was resuspended in membrane extraction buffer at 4°C for 10 min and centrifuged at 500 g for 5 min. This supernatant was used as a membrane extract.

### Immunoblot and Immunoprecipitation

Immunoblot analyses were performed as described previously [28]. Briefly, the cultured cells were lysed in lysis buffer containing 1% Nonidet P-40 (NP-40) and protease inhibitors, and insoluble materials were removed by centrifugation. The whole cell lysates (15 µg per each lane) or immunoprecipitated proteins were subjected to SDS–PAGE and electrophoretically transferred onto a polyvinylidene difluoride membrane (Immobilon, Millipore, Bedford, MA). After blocking the residual binding sites on the membrane, immunoblotting was performed with an appropriate Ab. For immunoprecipitation, protein extract was incubated with the appropriate Ab and protein A-sepharose beads at 4°C for 6 h. After SDS-PAGE, protein was transferred to a nitrocellulose membrane. The membranes were incubated in TBST blocking buffer (4% nonfat dry milk in Tris-buffered saline-Tween 20, 0.15 M NaCl, 0.01 M Tris-HCl pH 7.4, 0.05% Tween 20) followed by the incubation with the primary Ab at room temperature for 1 h. The primary Abs utilized in this study were as follows: anti-CALM (G-18), and anti-actin Abs (Santa Cruz Biotech., Santa Cruz, CA), anti-phoshotyrosine (4G10), anti-KIT (D13A2), anti-phosphorylated Akt (Ser473) (587F11), anti-Akt (C67E7), anti-phosphorylated p-44/42MAPK (T202/Y204) (20G11), anti-p44/42MAPK (ERK1/2) (137F5), anti-pan-Cadherin (28E12), anti-HSP90 (E289), anti-EEA1 (C8B1), and ant-LAMP1 (C54H11) Abs (Cell Signaling Technology). Then, the membranes were incubated with the appropriate secondary Abs diluted in blocking buffer, and immunoreactive proteins were visualized by enhanced chemiluminescence (LAS4010, GE Healthcare, Cleveland, OH).

### Statistical Analysis

Statistical analysis was performed with the Student t test. Error bars indicate the standard deviation (SD) of the mean. P-values less than 0.05 were considered statistically significant.

## Results

### Number of LSK Cells Decreases in Fetal Liver of *CALM* ^−/−^ Mice

**Figure 1:**
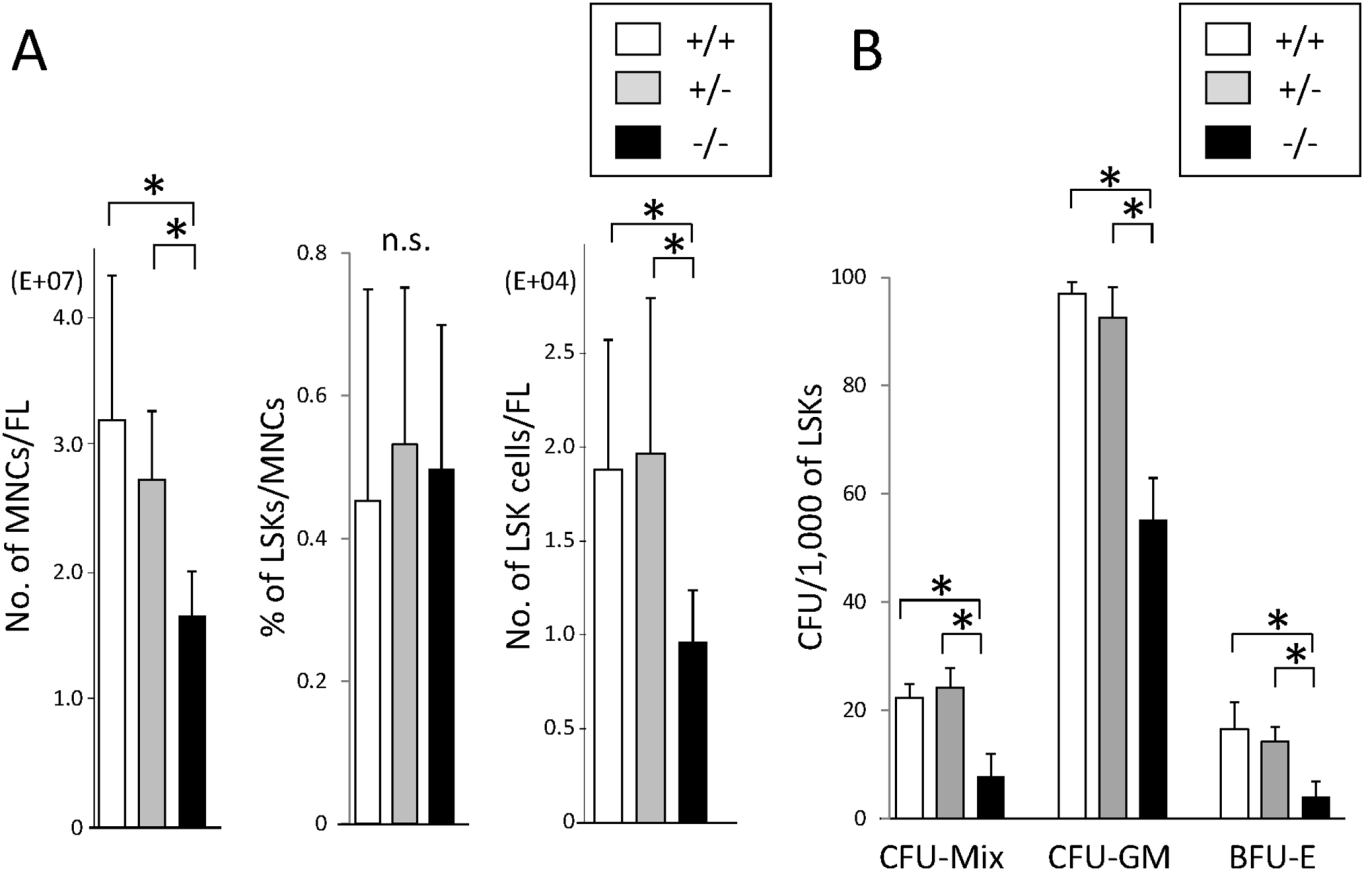
Hematopoietic stem/progenitor cells decreases in the fetal liver of *CALM* ^−/−^ mice and their colony forming activities are impaired. (A) Fetal liver cells were harvested from wild-type (WT) (indicated +/+), *CALM* ^+/−^ (indicated +/−), and *CALM* ^−/−^ (indicated −/−) mice on embryonic day 14.5 (E14.5). Numbers of mononuclear cells (MNCs) in each fetal liver were calculated by cell counter. The proportion of Linage^−^Sca-1^+^KIT^+^ (LSK) cells in MNCs was determined by flow cytometry. Data represent means ± SD (n = 6, *p<0.05). (B) LSK cells isolated from fetal liver of WT, *CALM* ^+/−^, and *CALM* ^−/−^ mice on E14.5 were subjected to colony assays. All cultures were conducted in triplicate and number of colonies were counted after 14 days. Results are shown as mean ± SD (n = 6, *p<0.05).

To analyze the role of CALM in hematopoietic stem/progenitor cells, we isolated fetal liver from WT, *CALM* ^+/−^, and *CALM* ^−/−^ mice on E14.5. Fetal liver from *CALM* ^−/−^ mice was macroscopically small compared with that from WT and *CALM* ^+/−^ mice. In accord with this finding, total number of hematopoietic MNCs significantly decreased in the fetal liver of *CALM* ^−/−^ mice compared with that in WT and *CALM* ^+/−^ mice (Fig. 1A, left). However, the proportions of LSK cells in MNCs were almost same regardless of their genotypes (Fig. 1A, middle). Thus, total number of LSK cells in the fetal liver of *CALM* ^−/−^ mice was about 50% compared with that in WT and *CALM* ^+/−^ mice. (Fig. 1A, right).

### Colony Forming Activity and Cytokine-dependent Growth Are Impaired in *CALM^−/−^* LSK Cells

We also performed colony assays by plating 1,000 LSK cells into the semisolid medium each containing the cytokine cocktail appropriate for the development of CFU-Mix, CFU-GM, and BFU-E. As shown in Fig. 1B, no apparent difference was observed in clonogenic activity between WT and *CALM* ^+/−^ LSK cells. In contrast, *CALM* ^−/−^ LSK cells yielded significantly less numbers of CFU-GM, BFU-E, and CFF-Mix than WT and *CALM* ^+/−^ LSK cells, indicating that colony forming activities were impaired in *CALM* ^−/−^ LSK cells. In addition, we found that the size of CFU (especially of CFU-Mix) formed from *CALM^−/−^* LSK cells was apparently smaller than that from WT and *CALM* ^+/−^ cells (Fig. S1). These findings indicate that *CALM* ^−/−^ LSK cells would have less activity to proliferate in response to cytokines.

**Figure 2:**
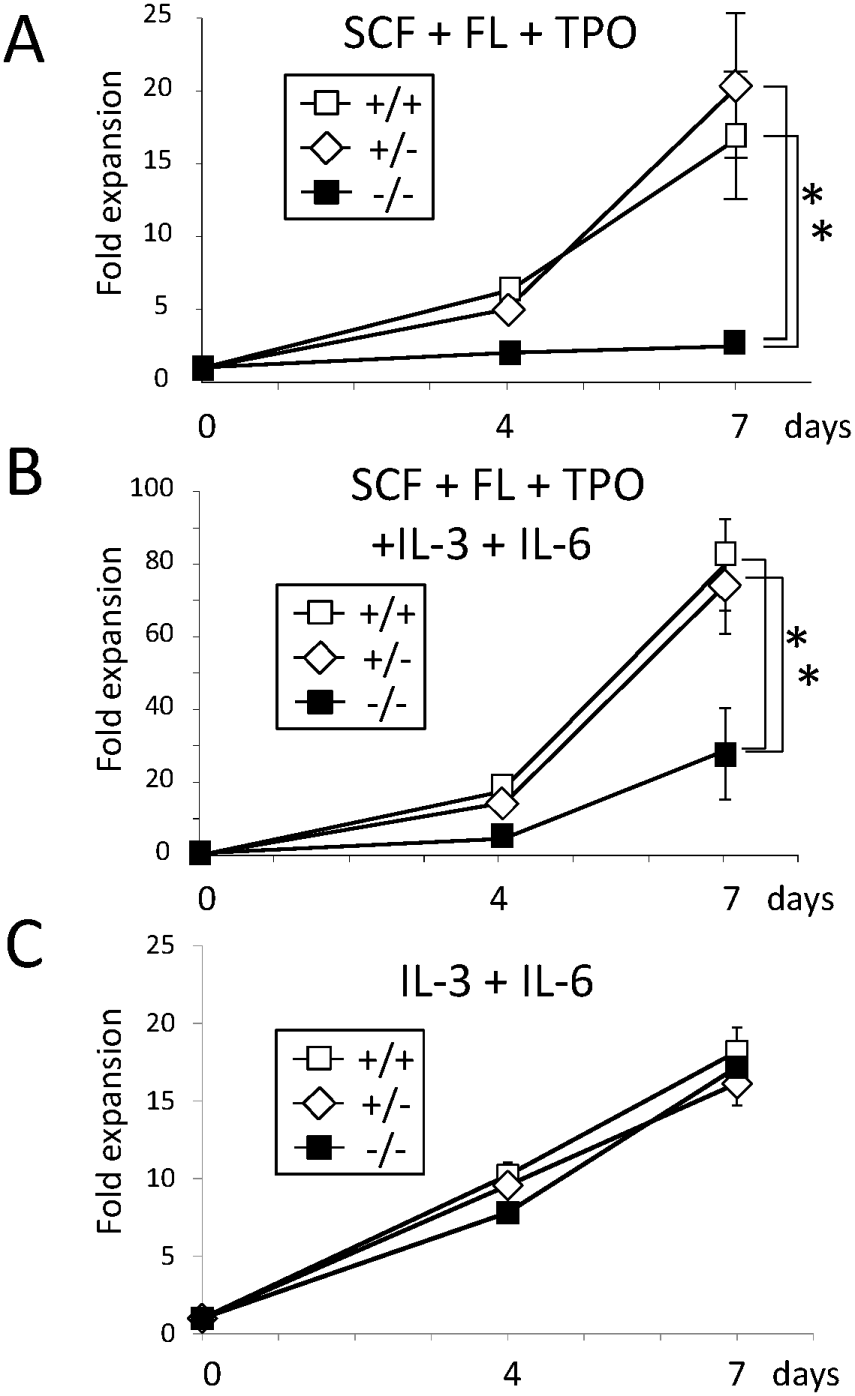
SCF, FL and TPO-dependent growth is impaired in *CALM* ^−/−^ LSK cells. (A) (B) (C) LSK cells isolated from fetal liver of WT, *CALM* ^+/−^, and *CALM* ^−/−^ mice were cultured in αMEM supplemented with 10% FBS in the presence of the indicated cytokines (100 ng/ml each). Total viable cell number was evaluated by the Cell Titer Glo Reagent (Promega) Data are shown as the mean ± SD of three cultures (n = 3, *p<0.05). SCF, stem cell factor; FL, Flt-3 ligand; TPO, thrombopoetin.

To confirm this hypothesis, we isolated LSK cells from fetal liver of WT, *CALM* ^+/−^, and *CALM* ^−/−^ mice and cultured them with SCF, FL, and TPO. As shown in Fig. 2A, the growth of *CALM* ^−/−^ LSK cells was apparently impaired compared with that of WT and *CALM* ^+/−^ LSKs. However, when IL-3 and IL-6 were added into this culture medium, the growth of *CALM* ^−/−^ LSK cells was not completely but partially recovered compared with that of WT and *CALM* ^+/−^ LSK cells (Fig. 2B). On the other hand, WT, *CALM* ^+/−^ and *CALM* ^/^ LSK cells show similar growth responses to IL-3 and IL-6 (Fig. 2C). These results indicate that CALM plays a crucial role in the transmission of growth signal from SCF, FL, and/or TPO but not from IL-3 or IL-6 whereas its haploinsufficiency doesn’t cause a clear defect.

### Endocytosis of SCF-bound KIT Is Not Impaired in *CALM* ^−/−^ MEFs

We next focused on the KIT signaling, because CALM has been reported to be involved in the internalization and/or intracellular transport of RTKs [29]. As cytoplasmic area of immature hematopoietic cells was rather small and not suitable for immunofluorescence analysis, we utilized MEFs from WT and *CALM* ^−/−^ mice, both of which were engineered to express KIT by the retrovirus infection.

At first, we confirmed that KIT was expressed at a similar level on the cell surface of WT and *CALM* ^−/−^ MEFs by measuring EGFP, which was expressed together with KIT from a single RNA by the bicistronic promoter of the retrovirus vector (data not shown).

**Figure 3:**
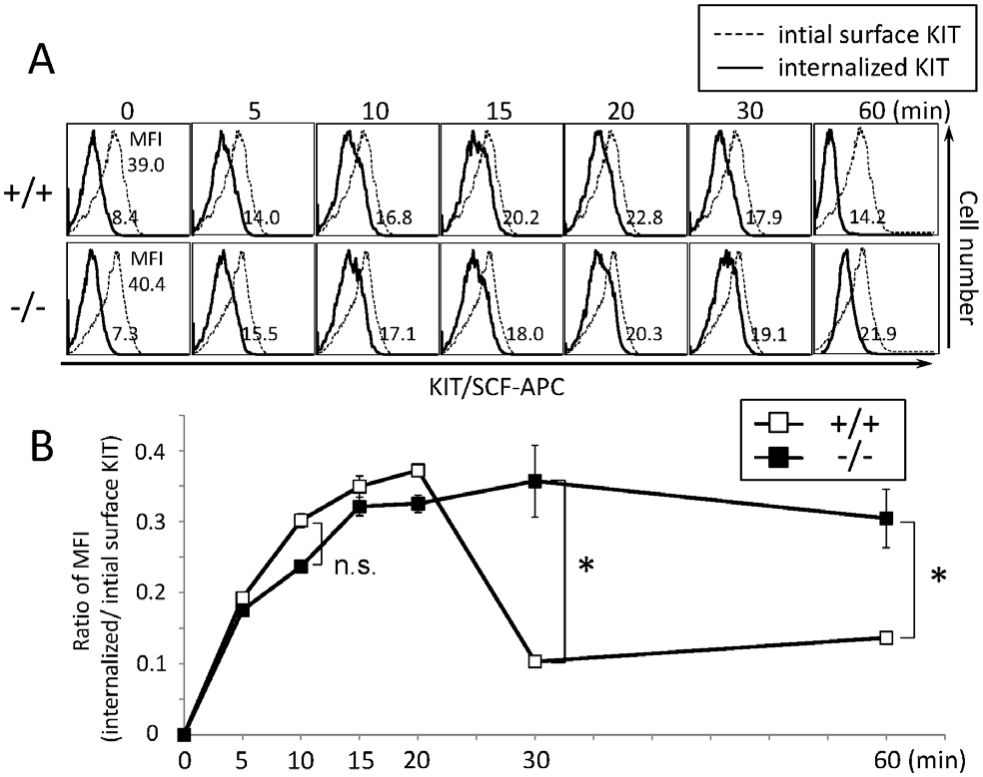
Endocytosis of SCF-bounded KIT isn’t affected by CALM deficiency. (A) WT and *CALM^−/−^* murine embryonic fibroblasts (MEFs) engineered to express KIT were incubated with biotinylated SCF for 60 min, and then with the APC-conjugated streptavidin for 30 min at 4°C. After stripping unincorporated SCF, the amount of the internalized SCF-KIT complex was quantified from the fluorescence intensity at the indicated times. (B) Uptake of SCF in WT or *CALM* ^−/−^ MEFs. The vertical axis indicates the ratio of mean fluorescence intensity, MFI (internalized SCF-KIT complex/initial surface KIT) (Data represent means ± SD, n  = 3, n.s.: not significant (p = 0.079)).

Next, we examined the internalization of KIT in WT and *CALM* ^−/−^ MEFs by incubating them with APC-labeled SCF at 37°C up to 60 min. After stripping unincorporated SCF, we quantified the amount of the internalized SCF from the intensity of fluorescence by FACS. As shown in Figure 3, after treatment with APC-conjugated SCF, the mount of SCF-KIT complex in the cytoplasm peaked at 20 min in both WT and CALM^−/−^ MEFs, indicating that the internalization of KIT wouldn’t be impaired by CALM deficiency. Then, the level of SCF-KIT complex decreased to the near basal level at 30 min and remained low up to 60 min in WT MEFs, suggesting that the internalized KIT was degraded and/or recycled in WT MEFs. On the other hand, it remained high up to 60 min in *CALM* ^−/−^ MEFs, implying that the intracellular trafficking of internalized KIT in *CALM* ^−/−^ MEFs would be rather different from that in WT MEFs.

### SCF-Induced KIT Trafficking from Early to Late Endosomes Is Impaired in *CALM* ^−/−^ MEFs

**Figure 4:**
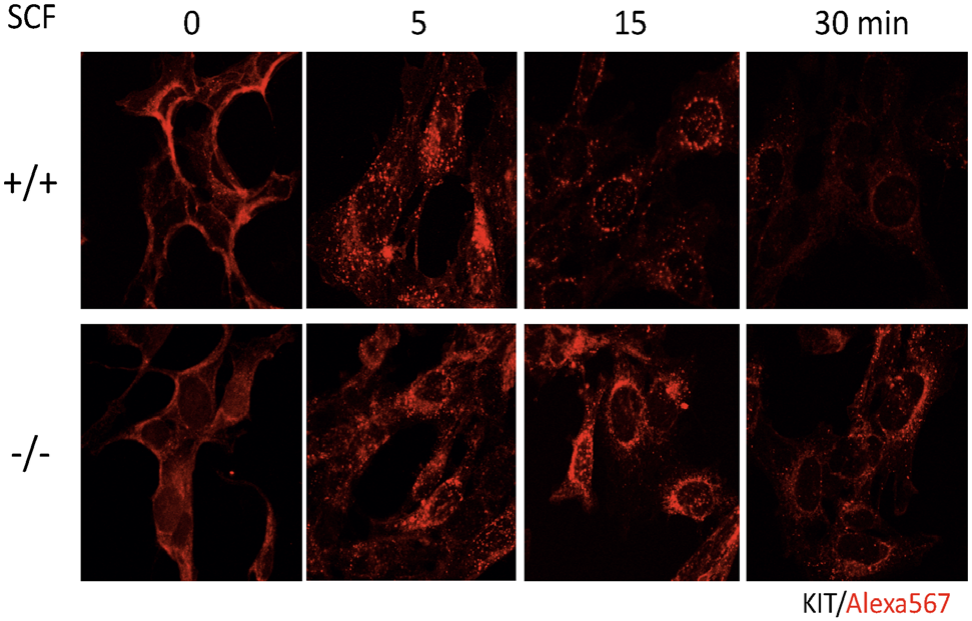
Localization of KIT after SCF stimulation is altered in *CALM* ^−/−^ MEFs. Localization of KIT was analyzed before and after SCF stimulation under confocal microscopy using WT and *CALM* ^−/−^ MEFs engineered to express KIT. KIT was visualized by the biotinylated anti-KIT antibody (Ab) and AlexaFluor 568 streptavidin conjugates.

Next, we analyzed intracellular KIT transport in WT and *CALM* ^−/−^ MEFs by immunofluorescence analysis. KIT was detected on cell surface prior to SCF stimulation and internalized into cytoplasm 5 min after SCF stimulation in both WT and *CALM* ^−/−^ MEFs (Fig. 4, upper and lower panels, time 0 and 5). Then, the internalized KIT adhered to the intracellular compartments surrounding nucleus, and subsequently formed a numerous punctate pattern in the cytoplasm in WT MEFs. Finally, the majority (about 80%) of KIT disappeared at 30 min in WT MEFs (Fig. 4, upper panel). In contrast, KIT was still easily detectable with about 80% of the basal level in *CALM* ^−/−^ MEFs at 30 min (Fig. 4, lower panel), indicating that the clearance of the internalized KIT from cytoplasm was impaired in *CALM* ^−/−^ MEFs.

**Figure 5:**
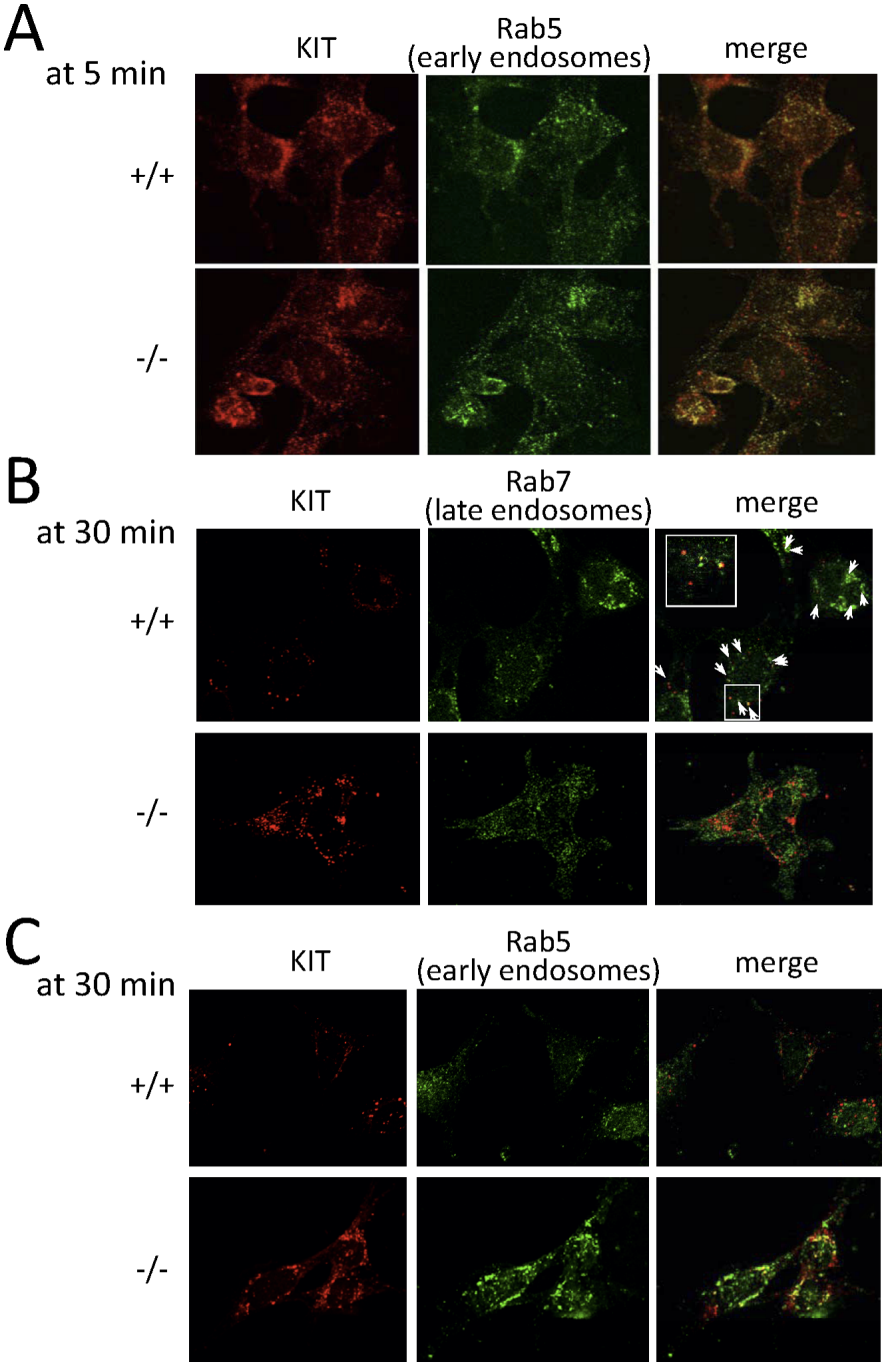
Intracellular trafficking of KIT from early to late endosome is impaired in CALM^−/−^ MEFs. (A) (B) (C) After SCF stimulation, intracellular localization of KIT was followed by immunofluorescence analyses with the anti-KIT Ab at the indicated times. Rab5 and Rab7 were used as markers of early and late endosome, respectively. Arrows indicate regions of colocalization (Inset shows region of higher magnification).

To analyze cytoplasmic localization of KIT in more detail, we utilized Rab5, Rab7, and Rab11 as a marker of early, late, and recycling endosome, respectively. At first, we confirmed that a significant proportion of CALM was colocalized with Rab5 at 5 min and with Rab7 at 30 min after SCF stimulation (Fig. S2). In accord with the localization of CALM, a substantial proportion of KIT was colocalized with Rab5 in both WT and *CALM* ^−/−^ MEFs 5 min after SCF stimulation (Fig. 5A), indicating that the transport of KIT to the early endosome isn’t disrupted in *CALM* ^−/−^ MEFs. Although only a faint signal of KIT was detectable in WT MEFs after 30-min SCF stimulation (about 20% of the basal level as shown in Fig. 4), the remaining KIT was colocalized with Rab7 in WT MEFs (Fig. 5B, upper panel). In contrast, KIT was scarcely colocalized with Rab7 in *CALM* ^−/−^ MEFs (Fig. 5B, lower panel). As for this reason, we found that KIT still remained in the early endosome in *CALM* ^−/−^ MEFs at this point (Fig. 5C, lower panel). On the other hand, CALM was scarcely colocalized with Rab11 in WT MEFs after SCF-treatment up to 30 min (the lowest panel in Fig. S2). Similarly, KIT wasn’t colocalized with Rab11 neither in WT nor in *CALM* ^−/−^ MEFs (Fig. S3). These results suggest that the internalized KIT isn’t recycled. These findings are in agreement with the previous report by Shimizu Y et al. [30], which indicated that new protein synthesis of KIT is required for its cell surface reappearance after SCF stimulation.

**Figure 6:**
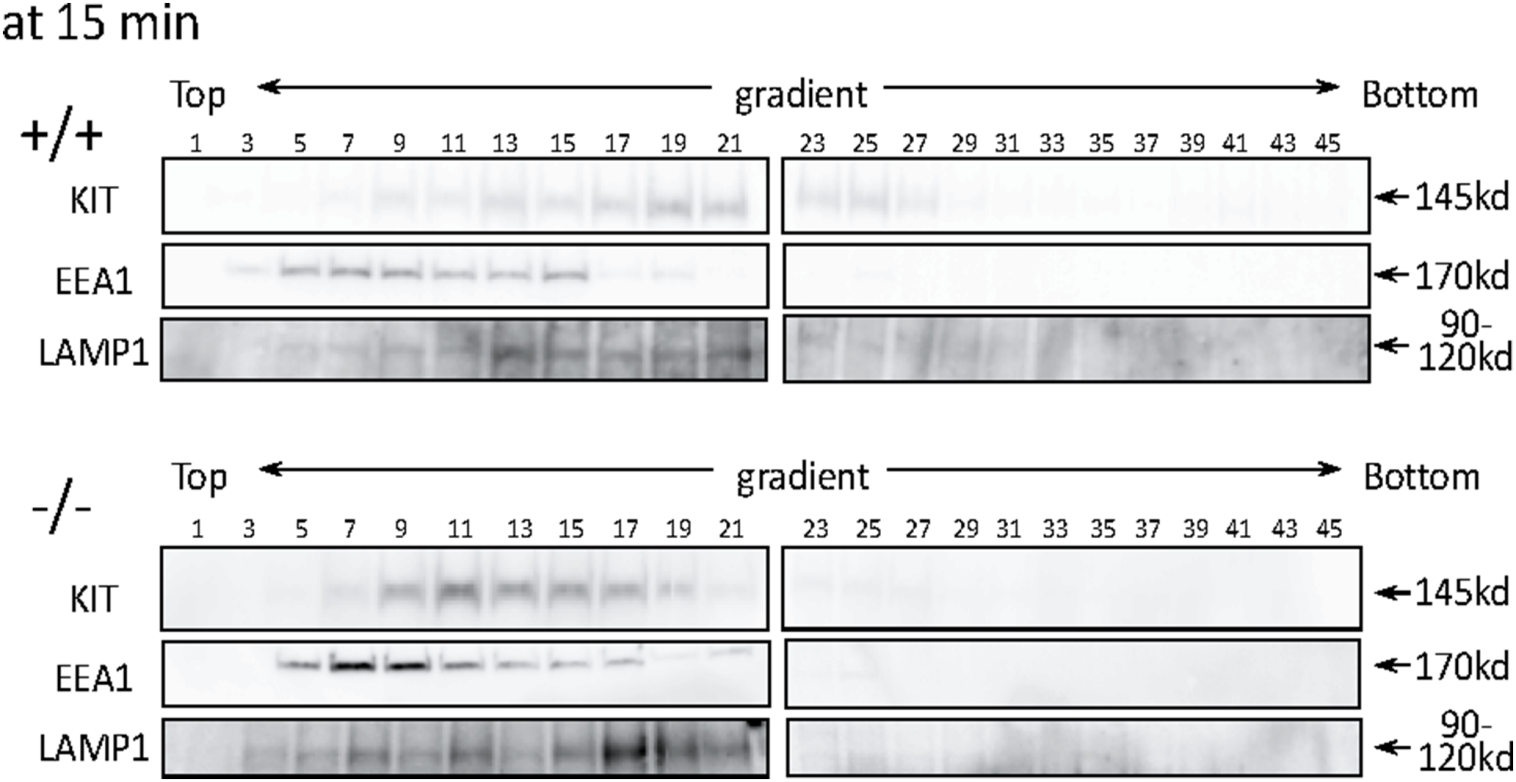
SCF-induced KIT trafficking from early to late endosome is impaired in CALM^−/−^ MEFs. Whole cell lysates were prepared from WT and *CALM^−/−^* MEFs after 15-min SCF stimulation. These lysates were fractionated with OptiPrep density gradient as described in Materials and Methods. Subcellualr localization of KIT was analyzed by immunoblot analysis with the anti-KIT Ab. EEA1 was utilized as an early endosome marker, and LAMP1 as a late endosome to lysosome marker.

To quantitatively demonstrate the difference in the intracellular localization of KIT between WT and *CALM* ^−/−^ MEFs after SCF treatment, we performed cell fractionation with OptiPrep density gradient. In this experiment, EEA1 was used as a marker of early endosome and LAMP1 as a marker of late endosome and lysozome, because these markers are more sensitive to discriminate each organella than Rab5 and Rab7 in this method. Treatment with SCF for 15 min led to the formation of a peak of KIT within late endosome to lysosome fractions (fractions 13–23) that are enriched in LAMP1 protein (Fig. 6). In contrast, although a small amount of KIT was detected in LAMP1-positive fractions (fractions 15–21), the majority of KIT was detected in EEA1-positive fractions (fractions 5–15) in *CALM* ^−/−^ MEFs. These results again indicate that SCF-induced KIT trafficking from early to late endosome was impaired in *CALM* ^−/−^ MEFs.

### SCF-induced Phosphorylation of Cytosolic KIT and Akt Is Enhanced and Prolonged in *CALM* ^−/−^ MEFs

**Figure 7:**
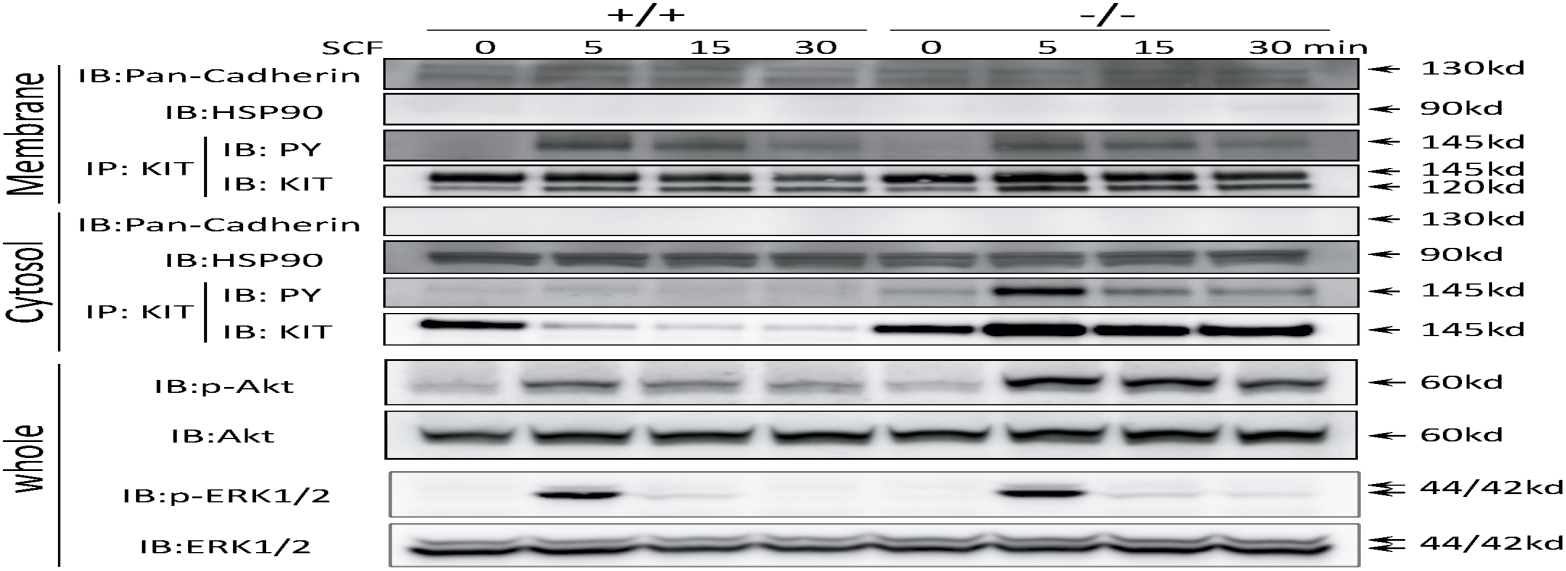
Diminution of cytosolic KIT after SCF stimulation is impaired in *CALM* ^−/−^ MEFs, leading to the enhanced and prolonged activation of KIT and Akt. WT and *CALM* ^−/−^ MEFs were stimulated with SCF and cellular lysates were isolated at the indicated times. Then, cytosolic and membrane fractions were separated, of which separation was confirmed by blotting with the Abs against pan-cadherin (reactive to only membrane fraction) and HSP90 (reactive to only cytosolic fraction). To examine the amounts and phosphorylation status of KIT, iimmunoprecipitated KIT proteins were subjected to immunoblot analyses with the indicated Abs. Phosphorylations of AKT and ERK1/2 were analyzed by immunoblotting using whole cell lysates.

We next examined whether KIT signaling was altered in *CALM* ^−/−^ MEFs because of the impaired trafficking from early to late endosome. For this purpose, we isolated membrane fraction (plasma, mitochondria and ER-Golgi membranes) and cytosolic fraction (containing endosomes) separately, of which separation was confirmed by the blotting with the Abs against pan-cadherin (reactive to only membrane fraction) and HSP90 (reactive to only cytosolic fraction). These lysates were subjected to immunoprecipitation and immunoblot analyses using the Abs indicated in Fig. 7. As shown in Fig. 7, 4th panel, both a mature (fully glycosylated, plasma membrane-bound, and functional) form and an immature (not fully glycosylated, membrane-unbound, and nonfunctional) form of KIT were detected in the membrane fraction with molecular weight 145 kDa and 120 kDa, respectively, of which levels were almost the same between WT and *CALM* ^−/−^ MEFs before SCF treatment. After 30-min SCF stimulation, the amount of mature KIT similarly decreased in both WT MEFs and *CALM* ^−/−^ MEFs (% decrease 17% and 13% by densitometric analysis, p = 0.67 (n = 3) (data not shown)). In addition, membrane KIT revealed similar phosphorylation pattern after SCF stimulation in both WT and *CALM* ^−/−^ MEFs (3rd panel). In contrast, although cytosolic KIT almost disappeared after 5-min SCF stimulation in WT MEFs, it was detected in *CALM* ^−/−^ MEFs (8th panel). This result seems to be inconsistent with our previous result obtained by immunofluoresence analysis indicating that KIT was still detectable in cytoplasm 5 min after SCF stimulation in WT MEFs (shown in Fig. 4, upper panel). As for this reason, we speculate that a substantial proportion of cytosolic KIT might be extracted as membrane protein because this fraction includes ER-Golgi membranes. Nonetheless, SCF-induced phosphorylation of cytosolic KIT (which is mainly located in early endosomes, Fig. 5A, 5B) was enhanced 5 min after SCF stimulation and prolonged up to 30 min in *CALM* ^−/−^ MEFs compared with that in WT MEFs (7th panel). In accord with this finding, SCF-induced phosphorylation of Akt was enhanced and prolonged in *CALM* ^−/−^ MEFs compared with that in WT MEFs (9th panel). In contrast, an apparent difference was not observed in ERK1/2 phosphorylation between these cells (11th panel). To confirm that the enhanced and prolonged phsphorylation of Akt observed in *CALM* ^−/−^ MEFs was dependent on KIT activity, we analyzed the effect of a KIT inhibitor, imatinib, on the activity of Akt after SCF stimulation in *CALM* ^−/−^ MEFs. As a result, we found that imatinib pretreatment inhibited both SCF-induced phosphorylations of KIT and Akt, implying that SCF-induced Akt phosphorylation was dependent on KIT activity (Fig. S4A). Furthermore, we confirmed that Bafilomycin A1, which inhibits protein transport from early to late endosome, enhanced and prolonged phosphorylation of KIT and Akt in SCF-stimulated WT MEFs as seen in *CALM* ^−/−^ MEFs (Fig. S4B). Taken together, these results indicate that KIT located in the early endosome can activate one of downstream molecules, Akt.

**Figure 8:**
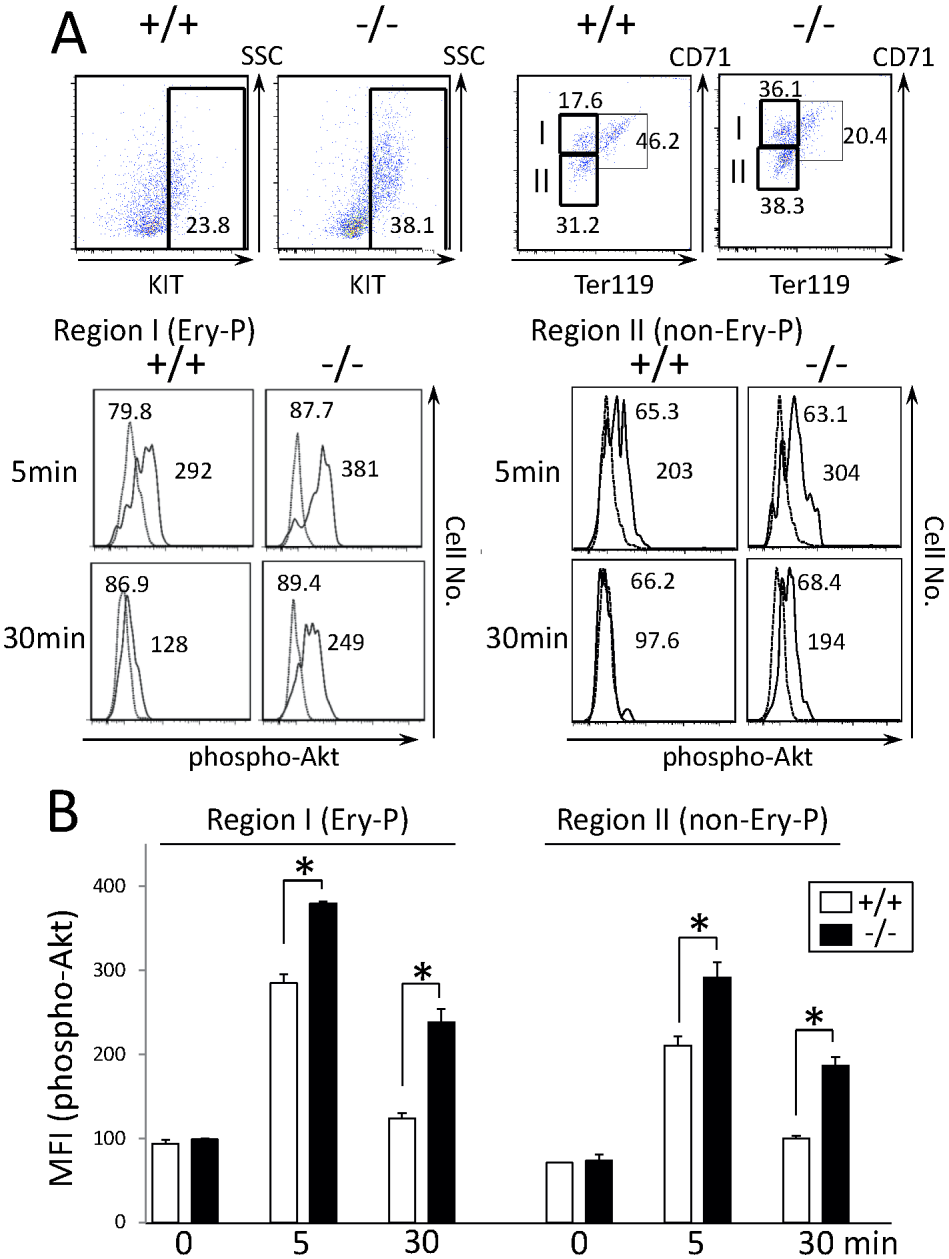
SCF-induced phosphorylation of Akt is enhanced and sustained in primary *CALM* ^−/−^ hematopoietic cells. (A) KIT^+^ fractions were compared between WT and *CALM* ^−/−^ fetal liver cells by flow cytometry. After the cultures without growth factors for 16 h, cells were stimulated with 100 ng/ml SCF for the indicated times. After permeabilization, Akt phosphorylation was analyzed with the anti-phospho-Akt (Ser-473) Ab by gating KIT+CD71^high^Ter119^low^ erythroid progenitor (Ery-P) fraction (Region I) and KIT+CD71^dim^Ter119^low^ non-erythroid progenitor (non-Ery-P) fraction (Region II). Unstimulated samples appear as a broken line in the FACS plots. (B) Mean fluorescence intensity (MFI) of phosho-AKT was quantified with an analytical tool, and the results are shown as the mean±SD (n = 3, *p<0.05).

Next, we analyzed whether KIT signaling was also altered in *CALM* ^−/−^ hematopoietic stem/progenitor cells. For this purpose, we isolated fetal liver cells from E14.5 WT and *CALM* ^−/−^ mice. As shown in Fig. 8A, KIT^+^ fraction increased in the total fetal liver cells from *CALM* ^−/−^ mice compared with that from WT mice (38.1% vs. 23.8%). We stimulated each fetal liver cells with SCF for the indicated times. Because cell components were different between WT and CALM^−/−^ KIT^+^ fraction, that is, the erythroid progenitor fraction was larger in CALM^−/−^ KIT^+^ fraction due to anemia than in WT KIT^+^ fraction (36.1% vs. 17.6%). So, we analyzed Akt phosphorylation in KIT^+^CD71^high^Ter119^low^ erythroid progenitor (Region I: Ery-P) fraction and KIT^+^CD71^dim^Ter119^low^ non-erythroid progenitor (Region II: non-Ery-P) fraction separately by gating with these markers. As shown in Fig. 8A and 8B, SCF induced excessive Akt phosphorylation to the similar extent in both erythroid and non- erythroid progenitors. This result indicates that the enhanced and prolonged Akt phosphorylation observed in CALM^−/−^ KIT^+^ fraction was not due to different cell components. Together, these results indicate that KIT signaling was altered in *CALM* ^−/−^ primary hematopoietic cells as well as in MEFs.

## Discussion

### Roles of CALM in Endocytosis and Intracellular Transport of KIT

Although cell surface interaction between SCF and KIT has been extensively studied [31]–[33], the precise mechanisms of their endocytosis and intracellular transport have not been clarified. Meanwhile, CME has been reported to be involved in the endocytosis and/or intracellular transport of some receptors such as epidermal growth factors (EGFs) [29], [34], vascular endothelial growth factor (VEGF) [35], [36] and Notch [37], [38]. So, in this study, we analyzed the roles of CALM in the endocytosis and transport of KIT. As a result, we found that CALM plays a critical role for the intracellular transport of KIT from early to late endosome, while neither endocytosis nor transport to the early endosome was affected by CALM deficiency. However, CALM has been shown to regulate proper formation of clathrin-coated vesicles in the process of clathrin-mediated endocytosis (CME), which is the major pathway of the internalization of ligand-bound RTKs, such as KIT and EGFR. So, our result that the internalization of KIT was not impaired by CALM deficiency seems to be strange. However, in agreement with our result, Kon S. et al. recently reported that the internalization of KIT was not impaired in the cells deficient for SMAP1, a member of the small GTPase Arf family, which is implicated in the formation of clathrin-coated vesicles as well as CALM [39]. So, we speculate that even if clathrin-coated vesicles might not be appropriately formed due to the deficiency of CALM or SMAP1, these deformed clathrin-coated vesicles could still work at the step of endocytosis of RTKs.

In addition, we found that the growth of *CALM* ^−/−^ LSK cells was severely impaired under the culture with SCF, FL, and TPO. In the current study, we focused on the KIT transport and its signaling. However, in our preliminary data, the transport of FLT3, which also belongs to the same class III receptor tyrosine kinase family, is also impaired in *CALM* ^−/−^ MEFs as was the case with KIT (data not shown). So, deregulated FLT3 signal may also be responsible for the impaired growth of *CALM* ^−/−^ LSK cells.

Activating mutations of KIT (KITV816F) and FLT3 (FLT3 internal tandem duplication), which are observed in about 10% and 8% of AML cases, respectively [40]–[42], have been considered to cause AML. Also, AML cases harboring these mutations are known to have poor prognosis [43]–[46]. Based on our data that CALM plays a critical role for the signaling from KIT and FLT3, it was expected that the manipulation of the CALM function would be an attractive new strategy to treat AML cases with such mutations.

### Activation of Downstream Molecules by KIT-containing Early Endosomes

Several previous papers demonstrated that PI3K/Akt signaling is confined to the plasma membrane and quickly lost once RTKs are internalized. In contrast, recent studies showed that some cytokine receptors and adaptor proteins located in endosomes can transduce their signals to downstream molecules, proposing the concept of “signaling endosome” [9]–[11]. In addition, it was also reported that oncogenic forms of Met and EGFR are mislocalized in cytoplasm, where they transmit aberrant signals to the downstream molecules. As for KIT, its recruitment to lipid rafts was shown to be necessary to activate PI3K/Akt [47]. However, it was also shown that WT and mutant KIT activate different sets of signaling pathways including Akt and MAPK due to their distinct intracellular localization. In addition, Xiang Z et al. reported that Golgi-localized oncogenic KIT can activate downstream molecules such as Akt, ERK, and STAT3 [48]. In this study, we found that SCF-induced phosphorylation of KIT in early endosomes was enhanced and prolonged in *CALM* ^−/−^ MEFs compared with that in WT MEFs, leading to the excessive activation of Akt. Similar results were also observed in *CALM* ^−/−^ KIT^+^ hematopoietic cells. These results indicate that KIT-containing early endosomes can indeed transit KIT signals to the downstream molecules as a signaling endosome. A very recent study showed that Bafilomycin A1, which inhibits protein transport from early to late endosome, enhanced FGF (fibroblast growth factor)-induced activation of ERK1/2 in HEK293 cells [27]. However, in contrast to Akt, we didn’t observe the enhanced phosphprylation of ERK1/2 (Fig. 7). So, it was speculated that the signaling pattern from early endosome would be somewhat different among RTKs.

### Significance of Enhanced and Prolonged Activation of Akt in *CALM* ^−/−^ LSK Cells

Many of the previous studies provided evidence that PI3K/Akt signaling is essential or important for the growth and survival in various cell types including hematopoietic cells [49]–[51]. In addition, constitutive activation of PI3K/Akt has been reported in AML cells, which is considered to participate in leukemogenesis and to be a good therapeutic target [52]–[54]. In contrast to these reports, in the current study, the impaired cytokine-dependent growth of *CALM* ^−/−^ LSK cells was accompanied by the enhanced and prolonged activation of Akt, suggesting that PI3K/Akt might be a negative regulator of cytokine-dependent growth and survival of hematopoietic cells. However, our result is supported by the fact that most of AML cells with Akt activity in the peripheral blood are in G1 phase of cell cycle [55]–[57], suggesting that constitutive activation of PI3K/Akt isn’t necessarily linked with cell growth. In addition, Akt activation was reported to induce premature senescence and sensitizes cells to ROS (reactive oxygen species)-mediated apoptosis [58]. Furthermore, decreased Akt phosphorylation is observed in a specific type of AML cells, in which the restoration of Akt activity promoted differentiation and disappearance [57]. Together, these results indicate that Akt can act as a both positive and negative regulator of cell growth and survival. To examine the relationship between the enhanced Akt activity and impaired growth of CALM^−/−^ hematopoietic cells, we inhibited PI3K/Akt activity by its inhibitors, LY 294002 and API-2 in CALM^−/−^ hematopoietic cells. In this experiment, we found that complete ablation of Akt activity by its inhibitor didn’t restore the growth but induced apoptosis in CALM^−/−^ hematopoietic cells (data not shown). These results suggest that Akt activity is essential for cell survival in hematopoietic stem/progenitor cells but appropriately regulated PI3K/Akt activity would be required for their full growth and survival.

## Supporting Information

Figure S1 **Picture of CFU formed from WT,** ***CALM^+/−^*** **, and** ***CALM^−/−^*** **fetal liver LSKs.** LSK cells isolated from fetal liver of WT, *CALM* ^+/−^, and *CALM* ^−/−^ mice on E14.5 were subjected to clonogenic assays. The size of colonies was observed under the fluorescence microscopy (BZ-X700, Keyence, Osaka, Japan). The representative results were shown. Mix, CFU-Mix; GM, CFU-GM; E, BFU-E. (+/+), (+/−), and (−/−) represent the origin of LSK cells: WT, *CALM* ^+/−^, and *CALM* ^−/−^ mice.
(TIFF)

Figure S2 **Distribution of CALM after SCF stimulation in WT MEFs.** Distribution of CALM was followed at the indicated times after SCF stimulation by immunofluorescence analyses using the anti-CALM Ab. Rab5, Rab7, Rab11 were used as markers of early, late, and recycling endosomes, respectively. Arrows indicate colocalization (Inset shows region of higher magnification).
(TIFF)

Figure S3 **Colocalization of KIT and Rab11, a marker of the recycling endosome.** KIT and Rab11 were costained with anti-KIT and anti-Rab11 Abs and analyzed by confocal microscopy.
(TIFF)

Figure S4 **Activation of downstream molecules by KIT localized at early endosomes.** (A) CALM^−/−^ MEFs were preincubated with 5 µM imatinib or vehicle before the treatment with SCF for 6 h. After SCF-stimulation, the amounts and phosphorylation status of KIT were analyzed at the indicated time points using immunoprecipitated cell lysates. Also, whole cell lysates were subjected to immunoblot analyses using anti-Akt and anti-phosho-Akt Abs. (B) WT MEFs were incubated with 1 µM Bafilomycin A1 or vehicle during the treatment with SCF and whole cell lysates were subjected to the same experiment as Fig. 7A.
(TIFF)
