# Supplementary figures and images for: Clathrin Assembly Protein CALM Plays a Critical Role in KIT Signaling by Regulating Its Cellular Transport from Early to Late Endosomes in Hematopoietic Cells
Source: PLoS One. 2014 Oct 3;9(10):e109441. doi: 10.1371/journal.pone.0109441 (PMC4184852; doi:10.1371/journal.pone.0109441)

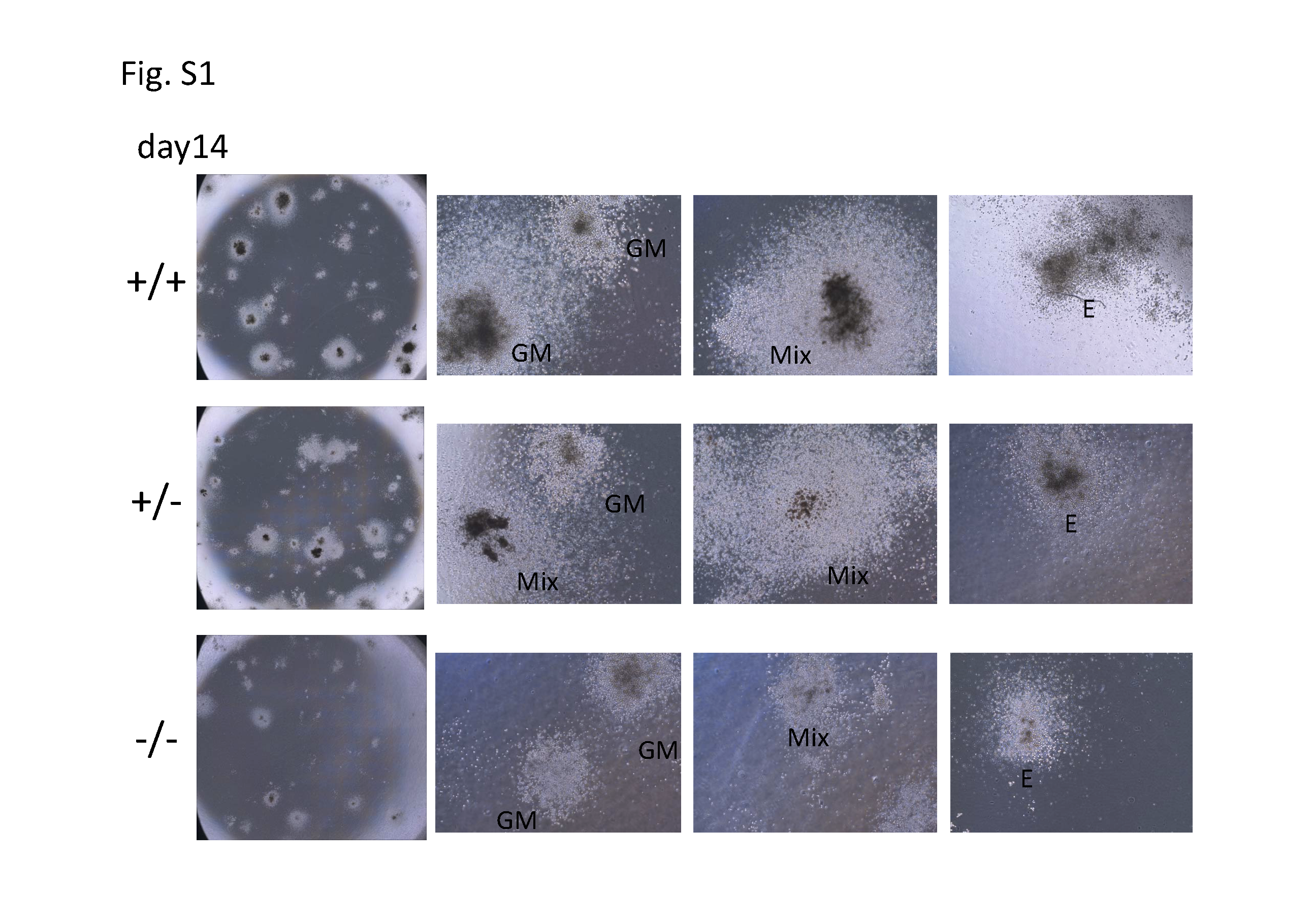

Supplement: Figure S1 — Picture of CFU formed from WT, CALM+/− , and CALM−/− fetal liver LSKs. LSK cells isolated from fetal liver of WT, CALM +/−, and CALM −/− mice on E14.5 were subjected to clonogenic assays. The size of colonies was observed under the fluorescence microscopy (BZ-X700, Keyence, Osaka, Japan). The representative results were shown. Mix, CFU-Mix; GM, CFU-GM; E, BFU-E. (+/+), (+/−), and (−/−) represent the origin of LSK cells: WT, CALM +/−, and CALM −/− mice. (TIFF) [file pone.0109441.s001.tiff]

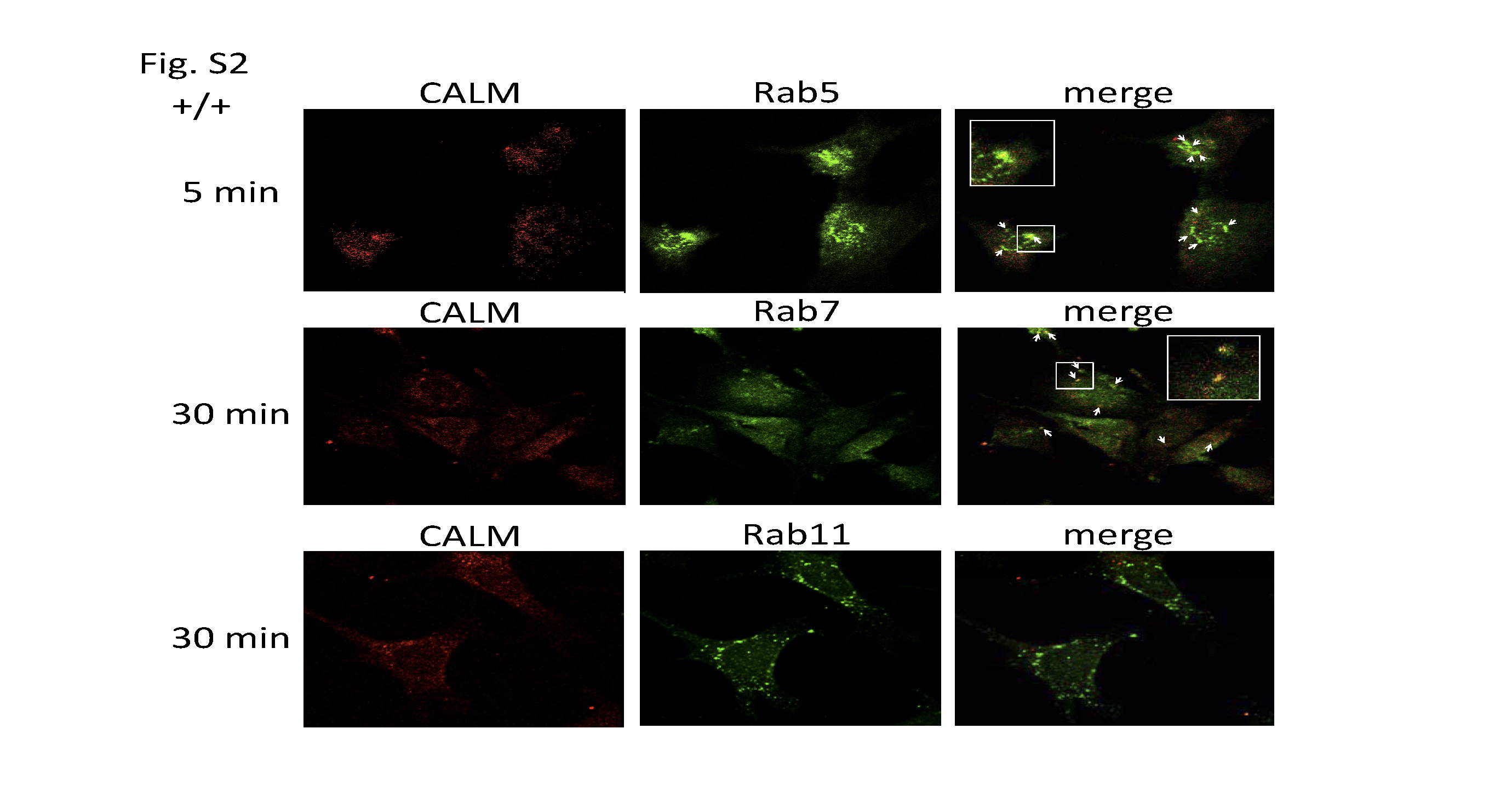

Supplement: Figure S2 — Distribution of CALM after SCF stimulation in WT MEFs. Distribution of CALM was followed at the indicated times after SCF stimulation by immunofluorescence analyses using the anti-CALM Ab. Rab5, Rab7, Rab11 were used as markers of early, late, and recycling endosomes, respectively. Arrows indicate colocalization (Inset shows region of higher magnification). (TIFF) [file pone.0109441.s002.tiff]

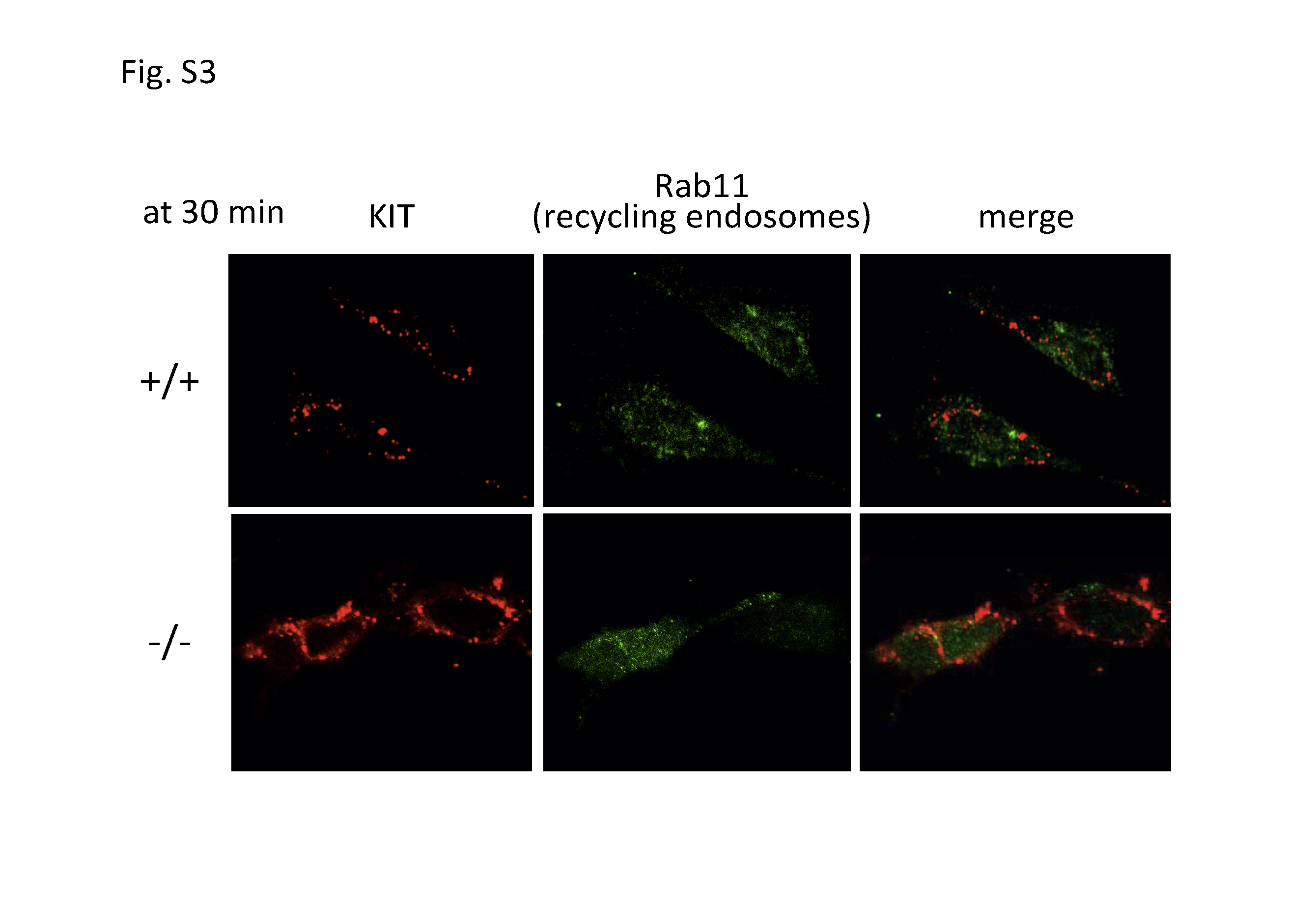

Supplement: Figure S3 — Colocalization of KIT and Rab11, a marker of the recycling endosome. KIT and Rab11 were costained with anti-KIT and anti-Rab11 Abs and analyzed by confocal microscopy. (TIFF) [file pone.0109441.s003.tiff]

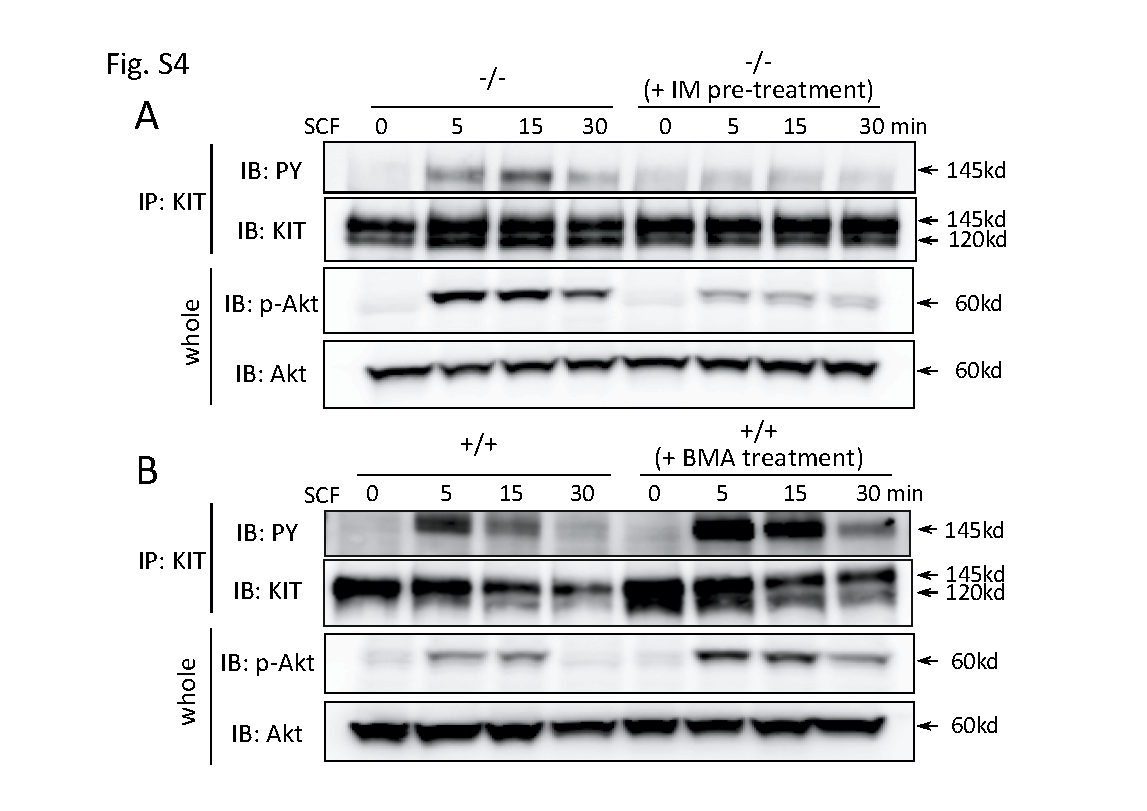

Supplement: Figure S4 — Activation of downstream molecules by KIT localized at early endosomes. (A) CALM−/− MEFs were preincubated with 5 µM imatinib or vehicle before the treatment with SCF for 6 h. After SCF-stimulation, the amounts and phosphorylation status of KIT were analyzed at the indicated time points using immunoprecipitated cell lysates. Also, whole cell lysates were subjected to immunoblot analyses using anti-Akt and anti-phosho-Akt Abs. (B) WT MEFs were incubated with 1 µM Bafilomycin A1 or vehicle during the treatment with SCF and whole cell lysates were subjected to the same experiment as Fig. 7A. (TIFF) [file pone.0109441.s004.tiff]
